# Evolutionary advantage of anti-parallel strand orientation of duplex DNA

**DOI:** 10.1038/s41598-020-66705-3

**Published:** 2020-06-18

**Authors:** Hemachander Subramanian, Robert A. Gatenby

**Affiliations:** 1 Department of Physics, National Institute of Technology, Durgapur, West Bengal India; 2 Integrated Mathematical Oncology Department, Cancer Biology and Evolution Program, H. Lee Moffitt Cancer Center and Research Institute, 12902 USF Magnolia Dr, Tampa, Florida USA

**Keywords:** Origin of life, Molecular evolution, Biological physics

## Abstract

DNA in all living systems shares common properties that are remarkably well suited to its function, suggesting refinement by evolution. However, DNA also shares some counter-intuitive properties which confer no obvious benefit, such as strand directionality and anti-parallel strand orientation, which together result in the complicated lagging strand replication. The evolutionary dynamics that led to these properties of DNA remain unknown but their universality suggests that they confer as yet unknown selective advantage to DNA. In this article, we identify an evolutionary advantage of anti-parallel strand orientation of duplex DNA, within a given set of plausible premises. The advantage stems from the increased rate of replication, achieved by dividing the DNA into predictable, independently and simultaneously replicating segments, as opposed to sequentially replicating the entire DNA, thereby parallelizing the replication process. We show that anti-parallel strand orientation is essential for such a replicative organization of DNA, given our premises, the most important of which is the assumption of the presence of sequence-dependent asymmetric cooperativity in DNA.

## Introduction

Living systems, uniquely in nature, acquire, store and use information autonomously. The molecular carriers of information, DNA and RNA, exhibit a number of distinctive physico-chemical properties that are optimal for storage and transfer of biological information^1–3^. This suggests that significant prebiotic evolutionary optimization^4^ preceded and resulted in RNA and DNA, and that the fundamental properties of nucleotides and DNA are not simply the outcomes of frozen accidents or of chemical inevitabilities. The evolutionary pressures that resulted in the adaptation of the specific physico-chemical properties of DNA are yet to be clearly elucidated, however. Such an evolution-based inquiry can be a useful alternative to the traditional biochemical approaches to unravel the functional significance of the structure and sequence of DNA. In this article, we identify an evolutionary advantage for the anti-parallel orientation of the two strands of the DNA duplex. The importance of such an evolution-based explanation for anti-parallel strand orientation^5^ stems from the fact that the latter is directly responsible for the biochemically cumbersome and complicated lagging strand replication mechanism of DNA, the existence of which militates against the well-established notion that DNA is a product of prebiotic evolutionary optimization. Evolution could have utilized parallel-stranded DNA, which have been shown to form under physiological conditions^6–10^, which could have obviated the need for lagging strand replication and the attendant biochemical complexities. The superior thermodynamic stability of anti-parallel DNA double strands over the parallel double strands cannot be a reason, since, in the primordial scenario, such a stability could actually have hindered self-replication by inhibiting the separation of daughter strand from the template^11^, and where the need for preservation of information is secondary or non-existent. Thus, the evolutionary choice of anti-parallel DNA as the genetic material requires explanation, given that parallel DNA double strands are proven to form within the physiological range of parameters, and, given the possible simplicity of self-replicative processes with parallel-stranded DNA. Within the picture we develop below, the evolutionary advantage of anti-parallel strand orientation of DNA arises from its ability to temporally parallelize the replication process, by dividing DNA into predictable, independent, simultaneously replicating segments, thereby speeding up the replication process considerably. In our picture, “Asymmetric Cooperativity”, a new property we introduced earlier and which we assume to be present in DNA, underpins the ability of anti-parallel strands to temporally parallelize DNA replication.

## On the Organization of the Article

The central concept of this article is asymmetric cooperativity, a new property of self-replicating heteropolymers that we introduced in our earlier article^12^. In that article, we have quantitatively evaluated, using a Marko Chain model, the self-replicative potential of heteropolymers with asymmetric and symmetric cooperativities. We have demonstrated there that heteropolymers with asymmetric cooperativity are evolutionarily superior, when compared to symmetrically cooperative or non-cooperative heteropolymers. The current article examines the evolutionary consequences of asymmetric cooperativity to the replicative organization of DNA. We begin below by recapitulating, from our earlier article^12^, what asymmetric cooperativity is and why it is useful for self-replication. In the next “Model and its Premises” section, we decompose asymmetric cooperativity into two parts, namely, sequence-independent and sequence-dependent asymmetric cooperativities, and elaborate on and illustrate them with a number of diagrams. We also explain the necessity of heteromolecular base-pairing, between purines and pyrimidines, to incorporate sequence-dependent asymmetric cooperativity, using a purely symmetry-based analysis. Literature-based experimental support, for our assumptions of asymmetrically cooperative bonding phenomena made in the “Model and its Premises” section, are provided in the “Experimental support for the model” section further below. We chose to sequester experimental support in a separate section in order to keep our model introduction as compact and comprehensible as possible, and to separate what is new in this article from what is already known. After the introduction of the model and its premises, in the next section, we logically demonstrate the evolutionary advantage of anti-parallel strand orientation, assuming the presence of asymmetric cooperativity in DNA. We also explore the possible emergence of a primitive kind of information storage in non-enzymatically self-replicating heteropolymers in the primordial regime, where information pertaining to the construction of enzymes was irrelevant. The sections following the “Experimental support” section are the “Falsification approaches” section, crucial for any testable scientific model, and the “Discussion” section, where a summary of our arguments are provided and the limitations of the model are underscored.

## Asymmetric Cooperativity

**Figure 1:**
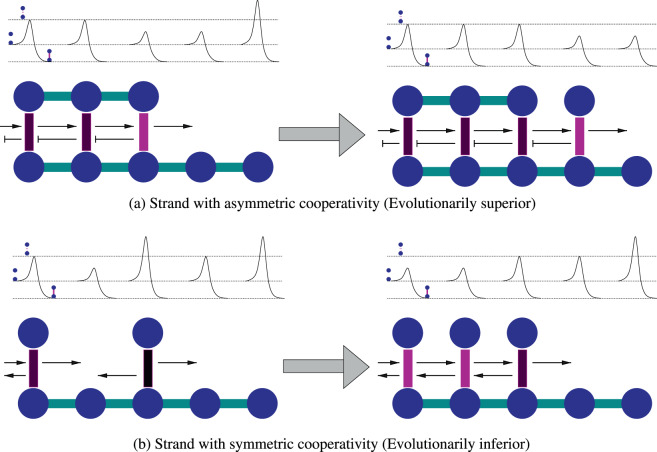
Illustration of replica strand construction process of a circular autocatalytic polymer in the presence of (**a**) asymmetric and (**b**) symmetric nearest-neighbor hydrogen bond cooperativities (reprinted from^12^ with permission from Elsevier). The circles represent monomers and the thick vertical lines connecting a pair represent the inter-strand hydrogen bonds. Horizontal lines connecting the monomers represent covalent bonds between them. The color of the hydrogen bonds represent the height of the kinetic barrier separating bonded and unbonded configurations, higher the barrier, darker the color. Hydrogen bonding energy diagram is shown above for both cases of cooperativity. The bottom line of the three lines in the energy diagram corresponds to the energy of the bonded configuration, and the middle line corresponds to that of the unbonded configuration. Only a section of the circular strand is shown here for convenience. (**a**) In a strand with asymmetric cooperativity, the catalytic influence on and from a hydrogen bond’s left and right neighbors are unequal, simplified here to be catalysis and inhibition from left and right, respectively. Catalysis from a neighboring hydrogen bond is denoted by an arrow from the neighboring bond, and inhibition, with a bar-headed arrow. Such non-reciprocal, asymmetric catalytic influence among a pair of neighboring hydrogen bonds leads to low kinetic barrier for hydrogen bond formation/dissociation near the growth front, enabling faster monomer utilization for the replica strand construction. This also prevents the pre-existing hydrogen bonds behind the growth front from dissociation and provides them longer lifetime to facilitate covalent bond formation between the replica strand monomers. This asymmetrically cooperative hydrogen bonding behavior solves the conundrum of simultaneously requiring both low and high kinetic barriers for hydrogen bond formation/dissociation stemming from the conflicting needs of high monomer utilization and high covalent bond formation probability, but renders the strands directional. (**b**) In strands with symmetric cooperativity, two hydrogen bonds mutually catalyze each other’s formation/dissociation, by reducing the kinetic barriers symmetrically. In this case, already formed hydrogen bonds in regions away from the growth front (second bond from left) have smaller kinetic barriers than the bonds at or near the growth front (fourth bond from left). This reduces the template’s ability to attract monomers for replica strand elongation, and also its ability to keep the monomers bonded long enough to facilitate covalent bond formation, thereby reducing its replicative potential relative to templates with asymmetric cooperativity. Please note that the scales of energies in (**a**,**b**) are not the same.

In an earlier article^12^, we showed that maximization of the replicative potential of a generic primordial self-replicating polymer leads to the property of asymmetric cooperativity. We recapitulate the same here for completeness. Asymmetric cooperativity is said to be present when the *kinetic* influence of a pre-existing hydrogen bond, between a monomer and the template strand of the polymer, on the formation/dissociation of the two neighboring inter-strand bonds between other monomers and the template, to the left and right, is unequal (please see Fig. 1). We theoretically showed that asymmetrically cooperative circular self-replicating polymer strands in the primordial oceans succeeded in the evolutionary competition with symmetrically cooperative self-replicating polymers for common substrates of their respective monomers and energetic sources. The advantage accruing to a generic circular self-replicating polymer from having asymmetric cooperativity is illustrated in Fig. 1. This replicative advantage of asymmetric cooperativity arises from the latter simultaneously satisfying two competing requirements for successful replication: A low kinetic barrier for a monomer to be easily inducted from primordial soup to form an inter-strand hydrogen bond, and a high kinetic barrier for the monomer to be retained on the template strand to facilitate intra-strand covalent bond formation in order to extend the replica strand. By lowering the kinetic barrier of its right (left) neighbor and raising the barrier of its left (right) neighbor, asymmetrically cooperative inter-strand bonds satisfy both these requirements, and result in a zipper-like functionality of the polymer, with unidirectional (un)zipping of inter-strand bonds.

It is obvious that there are two entirely equivalent *modes* of asymmetric cooperativity: *left asymmetric cooperativity*, where the kinetic barrier of the left neighboring inter-strand bond is lowered, and *right asymmetric cooperativity*, where the right neighbor’s barrier is lowered. Within the premise that DNA is a product of molecular evolution, it would be natural to expect that asymmetric cooperativity is present in DNA as well. In our previous publication^12^, we have suggested an experiment to verify the existence of asymmetric cooperativity in DNA, and cited numerous experiments suggesting its presence in DNA.

## The Model and its Premises

In this article, *our central premise is the presence of asymmetric cooperativity in DNA*. In order to simplify our arguments below, we factorize asymmetric cooperativity in DNA into two parts: A strong sequence-independent part, in which, the mode of asymmetric cooperativity (left or right) is dictated entirely by the orientation of the DNA *single strand*; and a comparatively weaker sequence-dependent part, where the mode is dictated by the “orientation” of the base-pair in the DNA *double strand*. The orientation of the base-pair specifies which nucleotide of the base-pair is on the 3′–5′ strand and which is on the 5′–3′ strand, thus differentiating, for example, the base-pair 5′–*G*–3′/3′–*C*–5′ from that of its 180°-rotated counterpart, 5′–*C*–3′/3′–*G*–5′. The kinetic effects on the left and right neighbors of a base-pair in these two orientations would be different, because of the base-pair’s left-right asymmetry. Below, we explain these two types of cooperativities in more detail.

### Sequence-independent asymmetric cooperativity

**Figure 2:**
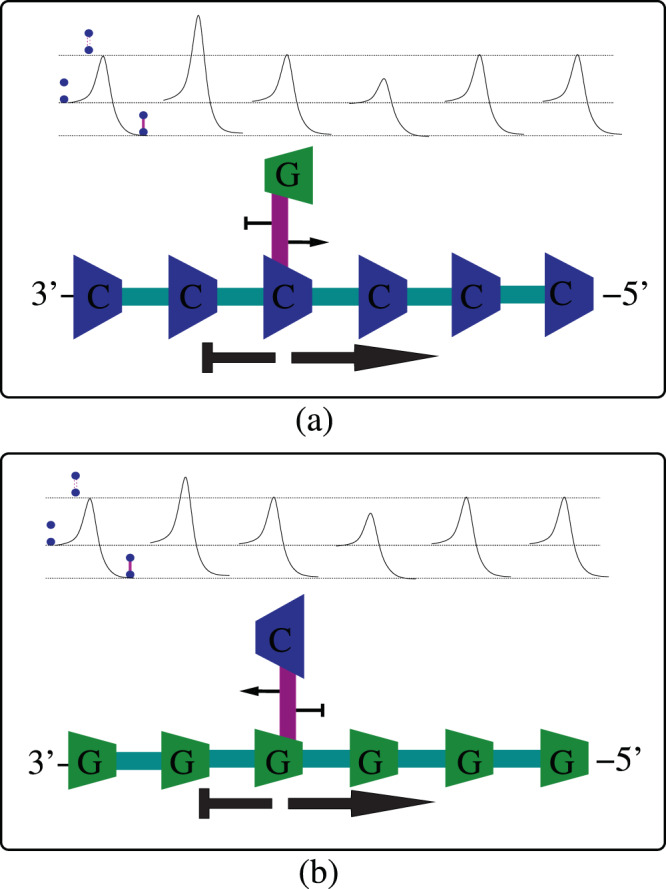
Illustration of sequence-independent asymmetric cooperativity in DNA single strands. The asymmetric cooperativity mode of a single template strand is dictated by the 3′–5′ directionality of the strand. (**a**) A hydrogen bond between a lone nucleotide $G$ and the template strand catalyzes the formation of another hydrogen bond to its right by reducing its kinetic barrier, while inhibiting the formation of its left neighbor by raising its barrier. The strength and the mode of sequence-independent asymmetric cooperativity dictated by the template strand is denoted by the thick black arrow below the template strand, pointing to the right. The thin arrows attached to the hydrogen bond denote the weaker sequence-dependent asymmetric cooperativity strength and mode. (**b**) Irrespective of the type of nucleotides occupying the template strand, the directionality of the template strand alone dictates the mode of sequence-independent asymmetric cooperativity. The thinner arrows on the hydrogen bond, denoting sequence-dependent part, though pointing in the opposite direction, does not alter the overall mode of asymmetric cooperativity due to the relative strength of sequence-independent part. This can be seen in the kinetic barrier diagrams above the bonds, where the barrier on the right of the hydrogen bond is lower in both (**a**,**b**), and the barrier on the left is higher. This assumption ensures that the daughter strand is always constructed from the 5′ end of the daughter strand to its 3′ end.

The sequence-independent asymmetric cooperativity mode is dictated by the orientation of the DNA *single strand template*: An interstrand hydrogen bond between a 3′–5′-oriented template strand and a lone 5′–3′-oriented nucleotide which is not yet incorporated into the growing daughter strand would catalyze its right neighboring hydrogen bond formation and inhibit its left neighbor (right asymmetric cooperativity mode). Reversing the template strand orientation from 3′–5′ to 5′–3′ would reverse the catalytic and inhibitory direction. Our theoretical separation of asymmetric cooperativity into a sequence-independent part and a sequence-dependent part implies that, in the case of the former, the asymmetric cooperativity mode is not influenced by the types of nucleotides composing the base-pair. Figure 2 illustrates the above point. The figure shows that, for a 3′–5′-oriented template strand, irrespective of the types of nucleotides composing the hydrogen bond, the kinetic barrier for the formation of a hydrogen bond neighbor to the right is always reduced, whereas, the barrier for formation of the left neighbor is always higher. The asymmetric cooperativity mode is the same in both the cases (a) and (b) in the figure, since the mode is dictated primarily by the directionality of the single template strand, denoted by the thick black arrows below the strands in the figure. Our assumption about the strength of sequence-independent cooperativity, in comparison with the weaker sequence-dependent cooperativity, leads to the former dominating the latter and dictating the asymmetric cooperativity mode in single template strands. Our above choice of the dependence of asymmetric cooperativity mode on the directionality of template strand ensures that the DNA daughter strand construction beginning at its 5′ end and moving towards 3′ end (towards the right in Fig. 2(a,b)) is kinetically favored, while construction beginning from the 3′ end of the daughter strand is disfavored. This premise is borne from the observation that DNA daughter strand construction is unidirectional and proceeds from its 5′ end.

### Sequence-dependent asymmetric cooperativity

**Figure 3:**
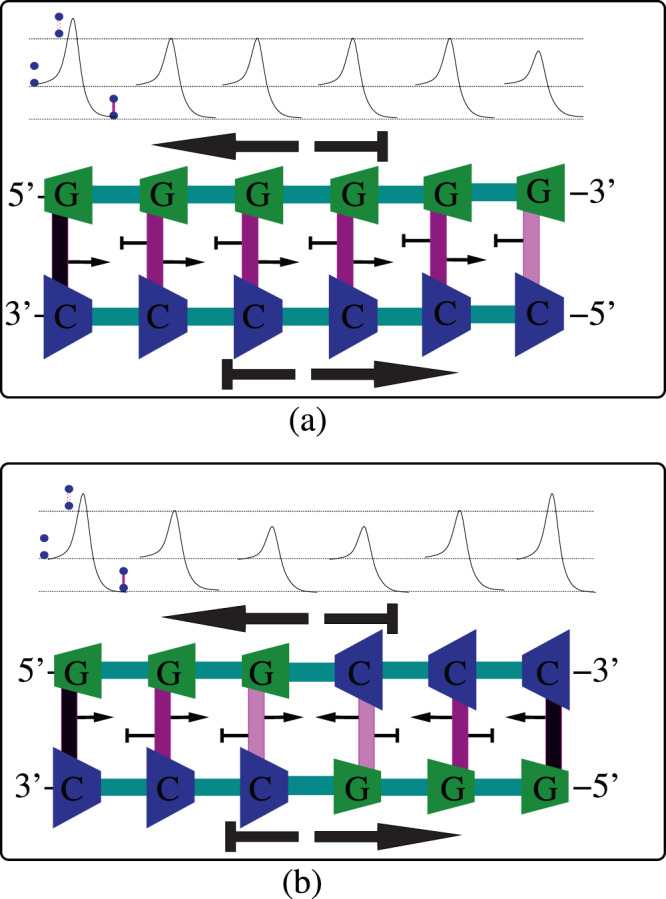
Illustration of sequence-dependent asymmetric cooperativity. In a DNA double strand, the anti-parallel orientations of the two strands result in the cancellation of their respective opposing asymmetric cooperativity modes. If the nucleotides on both the strands are of the same type, the cancellation would be complete, due to symmetry. When the types of nucleotides on the two strands are different, say, with *C* on the 3′–5′ strand and *G* on the 5′–3′, the cancellation is not complete and the residual asymmetric cooperativity is dictated by the types of nucleotides, making the asymmetric cooperativity sequence-dependent. The thick arrows denote the sequence-independent asymmetric cooperativity dictated by the individual strands’ directionality, whereas the thinner arrows attached to the hydrogen bonds denote the sequence-dependent asymmetric cooperativity that changes its mode depending on the orientation of the base-pair. 5′–*G*–3′/3′–*C*–5′ base-pair orientation of the hydrogen bonds instantiates right asymmetric cooperativity, as shown in (**a**), whereas the 180° -rotated 5′–*C*–3′/3′–*G*–5′ instantiates left asymmetric cooperativity, as the last three bonds of (**b**) illustrates. The kinetic barrier diagrams above the strands in (**a**,**b**) are significantly different, illustrating the sequence-dependence of the unzipping behavior of DNA double strands. The color of the hydrogen bonds represent the height of the kinetic barrier separating bonded and unbonded configurations, higher the barrier, darker the color. The unzipping of the strand in (**a**) would proceed sequentially from the rightmost end, whereas the middle two bonds would break and the strand will simultaneously unzip in both the directions in (**b**). This is proposed to lead to simultaneous construction of daughter strand on multiple segments of the single strand template in anti-parallel strands.

The sequence-dependent part of asymmetric cooperativity arises from the dependence of asymmetric cooperativity modes on the orientation of the base-pair. We assume that this sequence-dependent part is considerably smaller in magnitude compared to the sequence-independent part, in order to align our picture with the experimentally established behavior of DNA replication. The sequence-dependent asymmetric cooperativity is operative only in DNA double strands, *due to the mutual cancellation of the opposing sequence-independent asymmetric cooperativity modes of the two anti-parallel strands of the DNA double strand*. Figure 3(a,b) illustrate the impact of sequence-dependent part of asymmetric cooperativity on the hydrogen bond kinetic barriers. The thick black arrows in Fig. 3 denote the direction of the two sequence-independent asymmetric cooperativity modes (left or right), which align with the 3′–5′ direction of the strands, whereas, the thinner arrows attached to the hydrogen bonds denote the direction of the two modes of sequence-dependent asymmetric cooperativity. The base-pair 5′–*C*–3′/3′–*G*–5′ is assumed to be left-asymmetrically cooperative, as shown in the last three bonds of Fig. 3(b), catalyzing its left and inhibiting its right neighboring hydrogen bond, whereas the 180°-rotated 5′–*G*–3′/3′–*C*–5′ would obviously be right-asymmetrically cooperative, which would catalyze its right and inhibit its left neighbor. As can be easily seen from the Fig. 3, the kinetic barriers of different hydrogen bonds in parts (a) and (b) are very different, due to the difference in the sequences in the two subfigures. We will argue below that this sequence dependence of kinetics of unzipping is evolutionarily useful for the DNA, for, it provides the DNA with additional degrees of freedom to *modify its kinetics of unzipping (and hence self-replication) by modifying its sequence characteristics*. In Fig. 3(a), unzipping is kinetically favorable if it begins at the rightmost end, whereas, in Fig. 3(b), the unzipping would begin at the center of the strand and proceed bidirectionally to the left and right. Experimental support for our above choice of assigning right asymmetry mode to 5′–*G*–3′/3′–*C*–5′ comes partly from^13^, where, the kinetic influence on the nonenzymatic incorporation of neighboring nucleotides has been measured, which is reproduced with permission and elaborated on below as Fig. 10.

It has to be re-emphasized that, in our picture, while the asymmetric cooperativity mode of a hydrogen-bond between a lone nucleotide and the template strand is dictated primarily by the 3′–5′ or 5′–3′ orientation of the template strand, as illustrated in Fig. 2, the asymmetric cooperativity mode of a hydrogen-bond in a fully-formed duplex DNA is dictated by the orientation of the base-pair, as illustrated in Fig. 3. This is because, in the fully-formed duplex DNA, the opposite orientations of the two single strands result in cancellation of sequence-independent asymmetric cooperativity, due to their opposing modes, leaving the sequence-dependent asymmetric cooperativity of the base-pairs to dictate the kinetics of hydrogen bond dissociation of their neighboring base-pairs.

### Importance of heteromolecular base-pairing

**Figure 4:**
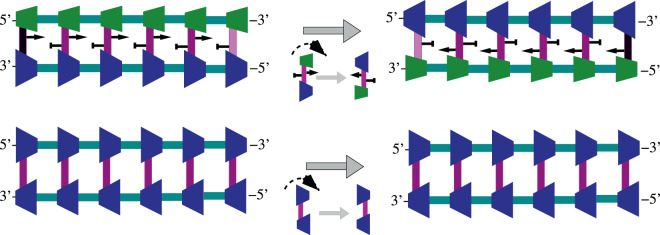
Illustration of the importance of heteromolecular base-pairing or base-pairing between different types of nucleotides. Incorporation of sequence-dependent asymmetric cooperativity requires the base-pair in anti-parallel DNA strands to be left-right asymmetric. On the top left and top right strands, base-pairs between different types of nucleotides, colored green and blue, are left-right asymmetric. Rotating the base-pair results in a directional structure with the blue and green nucleotides exchanged between the two strands, as shown below the arrow in the top center. Thus heteromolecular base-pairs can differentiate between their left and right, are directional, and hence can instantiate asymmetric cooperativity. Rotating the base-pair results in flipping of catalytic and inhibitory arrows associated with the base-pair and thus will impact the unzipping kinetics of the strand. It is easier to unzip the top-left strand from its right end, whereas, for the top-right strand, unzipping from its left end is kinetically favorable. This favorability is illustrated with the color of the hydrogen bonds, which represent the height of the kinetic barrier separating bonded and unbonded configurations, higher the barrier, darker the color. On the other hand, as the bottom left and bottom right strands illustrate, if the two nucleotides of a base-pair are of the same type, both colored in blue, Rotating the base-pair results in a non-directional structure, as shown below the bottom center arrow. Thus, homomolecular base-pairs cannot distinguish between left and right and hence cannot instantiate asymmetric cooperativity. Hence no catalytic or inhibitory arrows are shown in the bottom strands. Unzipping kinetics of the two bottom strands are the same due to their left-right symmetry.

It is important to note that, if not for the complementarity of the sequences of the two strands, left-right symmetry would prohibit the incorporation of asymmetric cooperativity in homomolecular base-pairs. This inability of homomolecular base-pairs to incorporate asymmetric cooperativity is illustrated in Fig. 4. Base-pairs such as 5′–*C*–3′/3′–*C*–5′, as shown in the bottom-left strand diagram of Fig. 4, are evidently *left-right symmetric*, cannot distinguish between left and right directions, and hence cannot instantiate asymmetric cooperativity. This can be verified by comparing the above base-pair structure with its self-similar 180° -rotated 5′–*C*–3′/3′–*C*–5′ structure, shown in the bottom-right strand diagram of Fig. 4. This is the reason no asymmetric cooperativity arrows are shown attached to the hydrogen bonds in the bottom-left and bottom-right strand diagrams of Fig. 4. Thus, in the fully formed anti-parallel DNA double strand, complementarity of the sequences of the two strands alone enable incorporation of asymmetric cooperativity, necessitating *heteromolecular base-pairing* and rendering the asymmetric cooperativity mode sequence-dependent. This ability to switch the mode of asymmetric cooperativity by rotating the base-pair is illustrated in the top-left and top-right strand diagrams in Fig. 4. If the DNA base-pairs are homomolecular, as illustrated in the bottom-left and bottom-right strand diagrams in Fig. 4, left-right symmetry of the duplex DNA base-pairs will disallow instantiation of sequence-dependent asymmetric cooperativity, while the sequence-independent asymmetric cooperativity would stand canceled due to the anti-parallel strand orientation of the daughter and template strands.

Our premise statements above are distilled from a number of experiments to help parsimoniously explain, using evolutionary reasoning, the counterintuitive replicative properties of DNA, such as its unidirectional daughter strand construction and the lagging strand replication mechanism, which are consequences of DNA’s anti-parallel strand orientation. We show below that these premises also help make sense of a few other disparate experimental observations such as the presence of asymmetric nucleotide composition or GC skew observed in nearly all genomes, and palindromic instabilities, apart from anti-parallel strand orientation of DNA. These premise statements about asymmetric cooperativity can be thought of as axioms or postulates, from which the replicative properties of DNA will be shown to follow logically. As postulates, these premise statements do not require biophysical justifications beyond the cited experimental literature that support their plausibility, in the “Experimental support” section below, and hence we defer an inquiry into the biophysical origins of these premises to a latter date.

Finally, we assume that the evolutionary force for faster construction of replica strand that was operative during the early stages of self-replicating polymer evolution was operative until more recently in guiding the evolution of various properties of DNA. Even though RNA is a more appropriate candidate to examine the consequences of asymmetric cooperativity, because it is widely believed to be evolutionarily more ancient than DNA, due to the comparative lack of experimental information on the thermodynamics and kinetics of double-strand formation and unzipping of RNA, and due to the central importance of DNA in understanding the functioning of extant biological systems, we decided to concentrate on DNA. More over, long RNA molecules are unstable in the extant biophysical environment, which renders the replicative organization of possible remnants of the “RNA world”, RNA viruses, uninformative, for our purposes. Due to this instability of long RNA molecules, the RNA viruses divide their genetic information across multiple, unconnected, short RNA molecules, called “segments”^14,15^, which also results in temporal parallelization of replication. The search for primordial biophysical environments that possibly enhanced the thermodynamic stability of long RNA molecules is ongoing^16,17^.

Continuing the reductionistic spirit of our earlier paper^12^, our intention here is to investigate the evolution of structural properties of DNA in isolation, without taking into account the effects of its interactions with numerous enzymes, such as polymerases. The rationale behind this assumption is that the fundamental properties of DNA, such as its anti-parallel strand orientation, were evolutionarily more ancient than the evolution of enzymes, and were already set by the evolutionary dynamics of the DNA’s progenitors before enzymatic assistance for replication evolved. The fact that such an inquiry throws much light on some of the counterintuitive properties of DNA justifies our approach *a posteriori*.

## Replicative advantages of anti-parallel DNA strands

The replicative advantage of anti-parallel DNA double strand arises simply from its ability to locally switch the modes of sequence-dependent asymmetric cooperativity from left to right or vice versa, since the stronger sequence-independent asymmetric cooperativity of the two anti-parallel individual strands cancel each other out. This switching of modes between left and right asymmetric cooperativity is achieved by altering the orientation of a hydrogen-bonded base-pair, by rotating it, as illustrated in the top-left and top-right strand diagrams in Fig. 4. For example, 5′–*G*–3′/3′–*C*–5′ base-pair orientation reduces the kinetic barrier of the hydrogen bonds to the base-pair’s right, thereby instantiating right asymmetric cooperativity mode, whereas the 180°-rotated 5′–*C*–3′/3′–*G*–5′ instantiates left asymmetric cooperativity, as shown in Fig. 3(b). As we show below, this sequence dependence of asymmetric cooperativity opens up the possibility of replicating a long DNA double strand by dividing it into multiple disjoint segments that are capable of replicating independently, simultaneously and predictably. These disjoint, independently replicating segments of DNA are called “Replichores” in Biology literature. This temporal parallelization of the replication process by dividing the DNA into multiple segments would have enhanced the replicative potential of the anti-parallel DNA double strand by significantly decreasing its replication time, compared to its biochemically distinct parallel strand self-replicating competitors^5,8,9^, during its early evolution.

**Figure 5:**
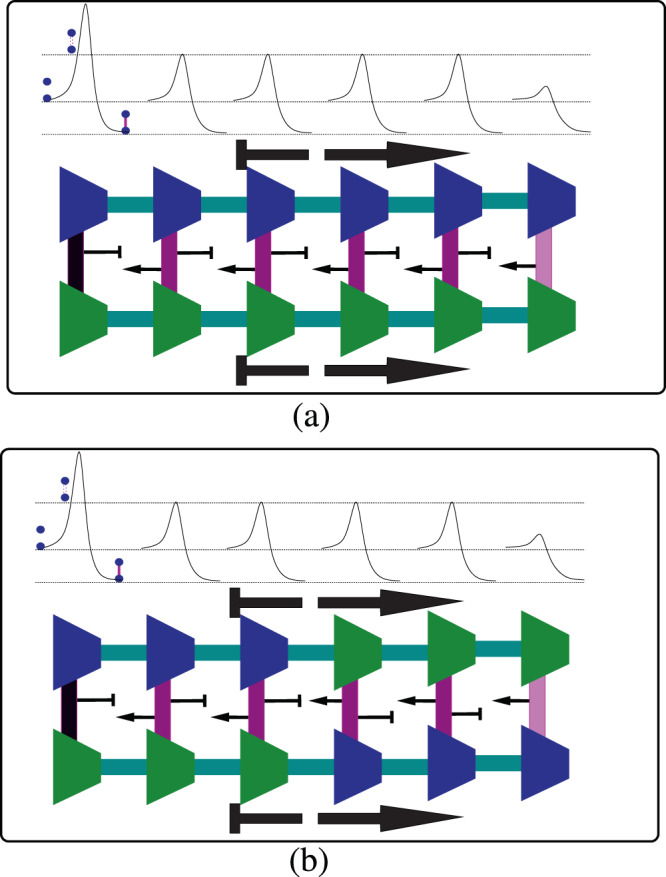
Illustration of the relative ineffectiveness of sequence variations in influencing unzipping kinetics in hypothetical parallel-stranded DNA-like molecule. The kinetic barriers for the interstrand hydrogen bonds, shown above the strand diagrams, can be seen to be similar in (**a**,**b**), irrespective of the difference in their sequences. Again, the color of the hydrogen bonds represent the height of the kinetic barrier separating bonded and unbonded configurations, higher the barrier, darker the color. This has to be contrasted with the substantially different kinetic barrier profiles between (**a**,**b**) of Fig. 3. The reason for the lack of influence of sequence on the kinetic barriers in the hypothetical parallel-stranded DNA-like molecule is because of the additive influence of sequence-independent asymmetric cooperativity of the two strands, which overwhelms the variations in kinetic barriers arising from the sequence-dependent part. Due to the cancellation of sequence-independent asymmetric cooperativity in anti-parallel double strand DNA, variations in the sequence results in strong variations in the kinetic barriers of interstrand hydrogen bonds. The unzipping kinetics of the anti-parallel DNA thus becomes intimately linked with its sequence, providing a crucial, additional degree of freedom.

The asymmetric cooperativity modes of the hypothetical parallel-stranded DNA-like molecule cannot be similarly altered locally, due to the predominance of the stronger sequence-independent asymmetric cooperativity over its sequence-dependent counterpart, arising from the directionally additive influence of the two parallel strands. This distinction can be understood by comparing the sequence-dependence of kinetic barriers of the hydrogen bonds of anti-parallel strands in Fig. 3 and the relative sequence-independence of kinetic barriers of parallel strands in Fig. 5. In Fig. 3, the heights of kinetic barriers of anti-parallel double strands are strongly dependent on the sequence, through the dependence of asymmetric cooperativity on the base-pair orientation. In Fig. 5, on the other hand, the kinetic barrier heights of hydrogen bonds of parallel double strands are relatively insensitive to the sequence, and is dictated primarily by the common orientation of the two parallel strands. This sequence-dependence of kinetic barriers arises in anti-parallel strands due to cancellation of sequence-independent asymmetric cooperativity because of the anti-parallel strand orientations of the two strands.

### Parallelization of the replication process

**Figure 6:**
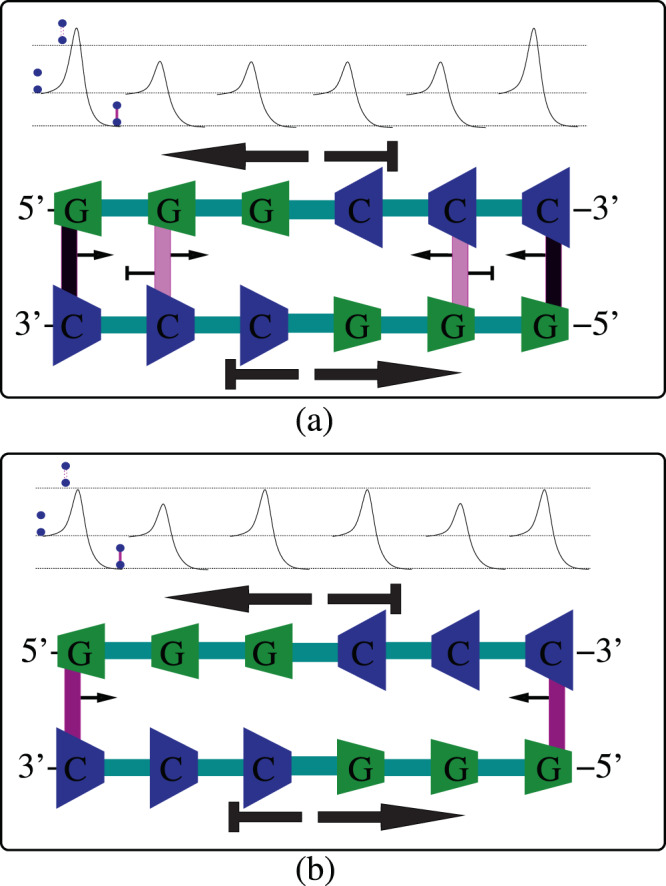
Parallel unzipping of the anti-parallel double strand DNA of appropriate sequence. As before, the color of the hydrogen bonds represent the height of the kinetic barrier separating bonded and unbonded configurations, higher the barrier, darker the color. Two hydrogen bonds in the middle of the double strand, between 5′–*GC*–3′/3′–*CG*–5′, reduce each others’ kinetic barriers due to dyadic symmetry of the sequence and are thus weaker than the rest of the bonds. Thus these two bonds will be the first bonds to break during temperature fluctuations, leaving their neighboring bonds on either sides with lower barriers due to the absence of their stabilizing influence. The weakening and dissociation of the hydrogen bonds thus propagate on either side of the “origin” of unzipping, simultaneously exposing two contiguous segments of the double strand to function as templates for the replica strand construction. Thus, with appropriate choice of the sequence, the anti-parallel DNA can parallelize its replication by simultaneously unzipping multiple segments and allowing for the construction of replica strand to proceed at multiple locations at once. The hypothetical parallel-strand DNA, on the other hand, would allow only for sequential unzipping from one of its ends where the kinetic barrier is lower. The additional degree of freedom offered by sequence variations cannot be utilized to parallelize replication in parallel-strand DNA, rendering the latter less evolvable.

**Figure 7:**
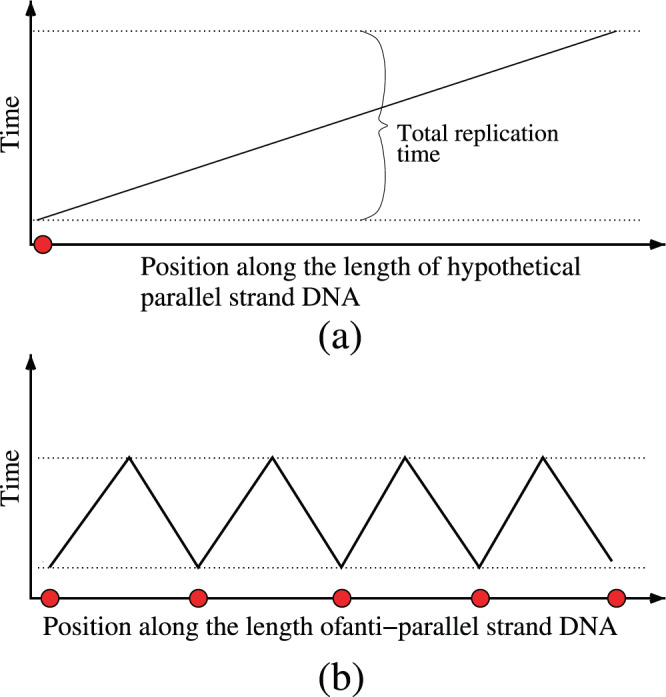
Schematic diagram illustrating the reduction in replication time of anti-parallel strands of appropriately chosen sequence compared to hypothetical parallel strand DNA. Points on X-axis denote the distance of an arbitrary location on the linear DNA strand from one of its ends. The schematic plots show the time at which a given location on the DNA is replicated. (**a**) In the hypothetical parallel-stranded DNA-like molecule, the double strand is constrained to unravel from only one of its two free ends, due to the lowering of kinetic barrier at that end through sequence–independent asymmetric cooperativity. This single origin of replication is denoted with a red dot on the X-axis above. Due to the sequential nature of unzipping of the parallel-stranded DNA-like molecule, locations farther from the origin would be replicated later, resulting in a replication time plot that increases monotonously with the distance from the origin. The straight line above schematically illustrates such a monotonically increasing replication time. (**b**) Anti-parallel double strand DNA offers the possibility of multiple origins, specified by sequences that have local dyadic symmetry, exemplified by palindromic sequences. These origins are denoted by multiple red dots on the X-axis. Disjoint segments are replicated simultaneously, and independently of each other, as shown by the multiple piecewise continuous lines representing each segment. These segments are called replichores in the literature. The replication time of anti-parallel strand DNA is reduced due to this parallelization of replication. Even though the sequence-independent asymmetric cooperativity of parallel-stranded DNA-like molecule is stronger, resulting in faster unzipping and hence possible faster movement of replication machinery along the strand, anti-parallel strands with slower rate of replication can overcome the competition by initiating replication at multiple locations, simply by acquiring sequences supporting multiple origins through evolutionary tinkering. To illustrate the above, the slope of the plot in (**a**) is drawn shallower, denoting faster rate of replication, compared to the slope of the plot in (**b**). It has to be noted that circular anti-parallel DNA strands need only one origin of replication to introduce two replichores or simultaneously replicating segments of DNA, and can reap the benefits of anti-parallel orientation.

The ability to switch the modes of asymmetric cooperativity between left and right by altering the sequences of the DNA in an anti-parallel DNA double strand makes it possible for independent segments of DNA to have different asymmetric cooperativity modes. This can be seen in Fig. 3(b), where the three left hydrogen bonds (left replichore) have right asymmetric cooperativity mode and the next three bonds (right replichore) are left asymmetrically cooperative. When the DNA begins to replicate, the earliest hydrogen bonds to break would be the ones with the lowest kinetic barrier, i.e., the third and the fourth bonds in Fig. 3(b), where the asymmetric cooperativity mode changes from right to left. This local unzipping process is illustrated in Fig. 6(a). The next two bonds to break would be the second and the fifth bonds, as shown in Fig. 6(b), whose barriers are lowered due to the absence of stabilization from the third and the fourth bonds, which were just broken. Thus the unzipping of the DNA double strand would proceed bidirectionally from the mode-switching location, as observed during DNA bubble formation before replication initiation in extant organisms^18,19^. This bidirectional unzipping from multiple such mode-flipping locations on DNA would make available multiple segments of DNA for simultaneous replication, unlike the hypothetical parallel DNA, where the unzipping would start at one end of the DNA (rightmost end in Fig. 5) and would have to proceed sequentially along the entire length of the DNA towards the other end to be kinetically favorable. This reduction in replication time of anti-parallel strands with appropriately chosen sequence is illustrated in Fig. 7. The Fig. 7(a) illustrates the sequential nature of unzipping and daughter strand growth in a hypothetical parallel strand DNA incorporating asymmetric cooperativity through a schematic diagram that shows the time at which each location on the double strand is replicated. It shows that the locations of DNA that are farther from the *origin of replication* (denoted by a red dot) are replicated latter, and there is a one-to-one correspondence between diffrent locations on the DNA and their time of replication. Figure 7(b) illustrates the parallel nature of replication in anti-parallel DNA strands with appropriately chosen sequence. Daughter strand construction radiating from multiple origins of replication (denoted by red dots), a consequence of sequence-dependent asymmetric cooperativity in anti-parallel DNA strands, creates disjoint segments that are replicated simultaneously, thereby reducing replication time. This reduction in replication time is robust even when the rate of daughter strand construction in anti-parallel strand is lower than that of parallel strand due to the smaller magnitude of asymmetric cooperativity, as illustrated by the higher slope of lines in Fig. 7(b). This robustness arises from the possibility of increasing the number of origins of replication and hence the number of segments, by appropriately choosing the sequences, thereby reducing the segment lengths and hence their replication time.

**Figure 8:**
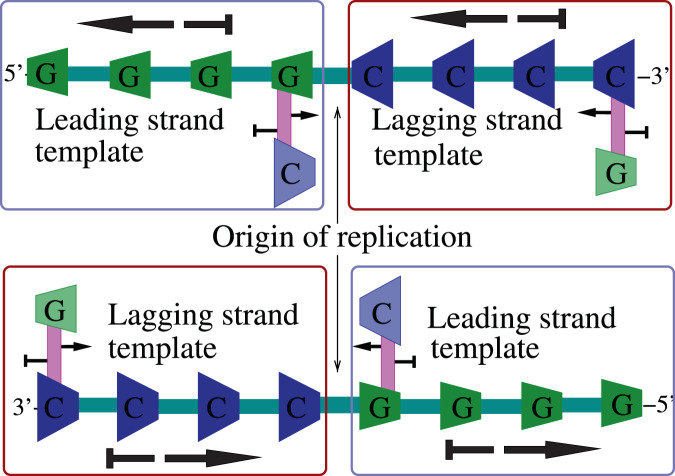
After the unzipping of a small section of anti-parallel DNA is complete, beginning from the “Origin of replication”, construction of the daughter strand begins on the exposed single strands. Sequence-independent asymmetric cooperativity dominates on single strands and the construction of daughter strand from the 3′-end of the template is strongly kinetically favored. Sequence-dependent asymmetric cooperativity, denoted by the arrows attached to the base-pairs in the diagram above, are rendered ineffective due to the presence of the stronger sequence-independent part, denoted by the thicker arrows along the strands. As the double strand unzips towards the left and right from the origin with temperature fluctuations, the template strands enclosed within blue boxes would allow the replication to proceed smoothly, since their 3′-end is located closer to the origin, from where the daughter strand construction begins and proceeds outwards, along the unzipping direction. These continously replicated template strands are termed “leading strand templates” in the biological literature. On the template strands where the direction of unzipping and the direction of daughter strand construction are opposite, as in the strands within the red boxes above, the daughter strand must necessarily be constructed discontinously. The construction can only begin after another burst of unzipping exposes enough single strand template, begins at the location farthest from the origin of replication at the 3′-end, and proceeds towards the origin. These template strands are called “lagging strand templates”. Asymmetric cooperativity helps to rationalize the non-intuitive replication behavior as the kinetically favorable option for DNA to replicate itself, through temporal parallelization of replication.

Once the DNA is locally unzipped bidirectionally, construction of daughter strands can begin anywhere on the two single-strand templates and proceed from the 3′-end of the template towards the 5′-end. But due to the sequence-independent asymmetric cooperativity of the single strand templates, the kinetically favorable replication initialization happens when the first hydrogen bond between the template and an incoming nucleotide is formed at the farthest of the unzipped 3′-ends of the two template strands, as shown in Fig. 8. In the Fig. 8, the lightly shaded *G* nucleotide denotes the location of the kinetically stable first bond formation on both the strands, beyond which the DNA double strand has not yet unzipped. As can be seen from this figure, the daughter strand construction can happen *continuously* on the template made available through unzipping only when the unzipping direction and the direction of the daughter strand construction are the same. This happens on parts of the two template strands labeled “leading strand templates” in the Fig. 8. When the direction of unzipping is opposite to that of the daughter strand construction, on parts labeled as “lagging strand templates” in the Fig. 8, daughter strand construction should begin at the farthest 3′-end made available by unzipping and proceeds towards the 5′-end, to be kinetically favorable. When another burst of unzipping happens beyond the initial bubble, the lagging strand construction should again begin at the farthest 3′-end of the recently unzipped template segment and proceed towards the 5′-end. In the extant organisms, the ingenious replisome design ensures that the RNA primers are attached to the lagging strand end closest to the helicase unzipping the DNA, and replicated from those ends discontinuously^20^. In the primordial settings that we are interested in, the Y-shaped fork itself might have catalyzed the daughter strand construction initiation at the 3′-end of the lagging strand template.

The picture we have developed thus far utilizes sequence-independent and sequence-dependent asymmetric cooperativities to argue that the experimentally observed DNA replication mechanism is kinetically the most favorable one. Furthermore, the above picture also suggests that the structural aspects of DNA, such as strand directionality and anti-parallel strand orientation, evolved to minimize the replication time and increase replicative potential.

### Information storage and sequence-dependent replication kinetics

We have argued above that the sequence characteristics of a primordial ancestor of DNA dictated its unzipping and replicative kinetics, through seuquence-dependent asymmetric cooperativity, instantiated by anti-parallel strand orientation and heteromolecular base-pairing. Sequences that support temporal parallelization of replication, through multiple alterations of the mode of asymmetric cooperativity between left and right, across the length of the polymer, such as 5′–(*G*)_*m*_(*C*)_*m*_(*G*)_*m*_(*C*)_*m*_–3′, for an arbitrary *m*, can successfully compete for monomers against a similar-length sequence such as 5′–(*G*)_4*m*_–3′, whose unzipping kinetics favor replication in a single file from right to left. The latter would take longer to replicate compared to the former (see Fig. 7). Thus, our hypothesis of sequence-dependent asymmetric cooperativity makes the connection between a specific sequence and its self-replicative potential in the primordial oceans, concrete. The competition for resources such as monomers, between different sequences, will result in certain sequences dominating over others in replicative potential, thereby giving rise to persistence of sequence properties, or information, across many cycles of replication of heteropolymers.

Environmental conditions, such as the abundance of monomers, temperature, pH and so on, would influence the rate of replication, and hence would also influence the type of sequences that would be successful in a given environment. For example, when monomers are highly abundant, sequences such as 5′–(*G*)_*m*_(*C*)_*m*_(*G*)_*m*_(*C*)_*m*_(*G*)_*m*_(*C*)_*m*_–3′ would replicate faster than the sequence with the same length 5′–(*G*)_*n*_(*C*)_*n*_(*G*)_*n*_(*C*)_*n*_–3′, with *n* > *m*, due to the presence of more independently replicating subunits in the former. Whereas, when the monomer supply is scarce, sequences that kinetically promote the retention of monomers bound to the template, and avoid multiple origins of replication which require multiple, simultaneous daughter strand construction initiations, such as the latter, will be more successful in replication. Thus the environment would influence the type of sequences that will be successful in it, leaving a crude imprint of itself in the sequences. The origin of information storage and processing in living systems is usually argued to be when an RNA or its ancestral self-replicator began forming a sequence-dependent three-dimensional folded structure that catalyzed the self-replication of itself and of its hypercyclic partners^21^. Here, we argue for the possibility of existence of heteropolymers whose replicative success in a given environment depend on their sequences, through sequence-dependent unzipping kinetics, leading to a more primitive form of information storage in the sequences that reflects the kind of environment in which they would succeed.

## Experimental support for the model

Multiple, independent lines of experimental observations in the literature, when reinterpreted, support the central thesis developed above, that the kinetics of unzipping during the replication/transcription of DNA depends on the sequence through sequence-dependent asymmetric cooperativity. Observations, such as the pervasive presence of asymmetric base composition or *GC* skew in nearly all genomes studied, which has resisted a simple explanation thus far, finds a surprisingly simple explanation within the model developed above. Furthermore, the observations of polar inhibition of the replication forks, palindromic instability and primer extension kinetics lend support to the existence of sequence-dependent asymmetric cooperativity. Below, we list these various experimental observations and elaborate on how they support our thesis.

### The presence of asymmetric nucleotide composition or *GC* skew

**Figure 9:**
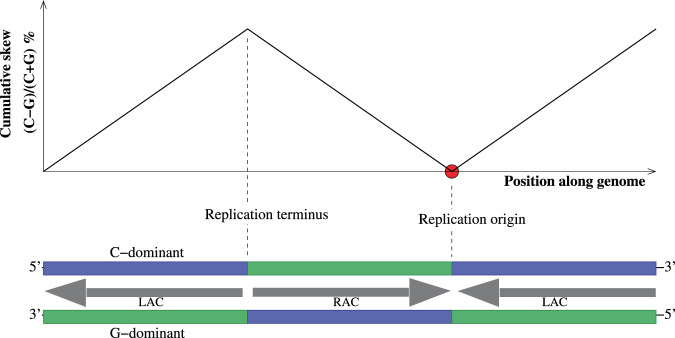
Schematic diagram illustrating the experimental observations related to *GC* skew in various genomes. The genome is composed of independently replicating subunits, usually refered to in biological literature as “replichores”, three of which are shown here colored in blue and green. The replichore that is enriched in *C* is denoted in blue, and in *G*, green. The sign of *GC* skew has been observed to correlate with the direction of replication along a template strand. Leading strand templates, the segments where replication and unzipping machineries travel in the same direction, have been observed to be enriched in the nucleotide *G*. Lagging strands, where these two machineries travel in opposite direction, must then be enriched in *C*. It has also been observed that only one of the boundaries between the replichores function as origin of replication, whereas the other, as replication terminus. The schematic graph above the strands illustrate the cumulative *GC* skew, calculated in running windows of appropriate size over the entire genome. It is representative of skews observed in genomes of multiple species, and clearly shows the boundaries between replichores. The *GC* skew has traditionally been attributed to the difference in replication mechanisms between the leading and lagging strands. All these observations are understandable within our picture of sequence-dependent asymmetric cooperativity, where *GC* skew is treated as the *cause* of unzipping directionality. The arrows between the two strands, labeled RAC and LAC, denote the right and left modes of sequence-dependent asymmetric cooperativity respectively. At the origin of replication, pointed by a red dot on the graph above, the asymmetric cooperativities reduce the barrier from both left and right, rendering the bonds at the interface weaker and thereby allowing the interface to function as the origin. At the terminus, the barrier height for the hydrogen bonds are raised from both directions and can be broken only when the neighboring bonds are broken.

Asymmetric base composition or *GC* skew, defined as a *local* excess of *G* over *C* or vice versa in one of the strands of the duplex DNA, has been observed in nearly all genomes studied, both prokaryotic and eukaryotic^22–27^. This strand asymmetry, calculated as $(C-G)/(C+G) \%$ in running windows along genomic sequences, can be positive or negative at different locations, and its magnitude averages to about 4% in Human genome^28^ and is more than 12% in some Bacteria^29^. The characteristic signature of the presence of $GC$ skew is a “V”-shaped cumulative skew diagram, as illustrated in Fig. 9. *GC* skew is traditionally used in genome analysis software programs to find “Origins of Replication” in prokaryotic genomic sequences, by identifying locations on the 5′–3′ strand where the skew switches from $G$-dominant to $C$-dominant. Various reasons have been provided for the presence of $GC$ skew in genomes, with the most prominent one attributing it to the asymmetric mutational pressures due to the differences in leading and lagging strand replicative and transcriptional mechanisms^30–32^, while the relative magnitudes of the mutational pressures due to replication and transcription still remain contentious^33,34^. Again, this reasoning does not provide the evolutionary significance of $GC$ skew, but only provides the mechanistic reason for its emergence. The question of the evolutionary advantage of $GC$ skew is important, because, higher the $GC$ skew, lower will be the space available for coding amino acids. For example, if there are very few or no .*G*’s available on a part of the transcribed DNA strand, due to very high $GC$ skew, then the DNA codons that have $G$ in them, such as 5′–*CTG*–3′, cannot be used to code for the amino acid *Leucine*, forcing the organism to code for the amino acid using other synonymous triplets, such as $CTA$. Thus $GC$ skew places restrictions on the redundancy of the Genetic Code, and hence is possibly detrimental, making its evolutionary significance much more intriguing.

The model we described above provides both the mechanistic and evolutionary underpinnings of $GC$ skew. The significance of $GC$ skew is apparent from the Fig. 9. The figure clearly illustrates our idea that the skew is the *cause* of direction of unzipping during DNA replication. The duplex strand shown in Fig. 9 shows three replichores, which are the independently replicating segments of DNA, oriented in such a way that the first segment is left asymmetrically cooperative, the second, right, and the third, left asymmetrically cooperative, again. Since left asymmetric cooperativity is instantiated by 5′–*C*–3′/3′–*G*–5′ as shown in Fig. 3(b), the first segment to the left is composed of 5′–3′ top strand that is C-dominant, and 3′–5′ bottom strand that is G-dominant. Similarly, for the right asymmetrically cooperative duplex strand, the 5′–3′ top strand is $G$-dominant, and 3′–5′, $C$-dominant. On a side note, an objection may be raised because the experimentally observed excess of the *G*– or *C*– dominance is only of the order of a few percent. This objection can be addressed by relaxing the assumption in our model, that the kinetic effects of asymmetric cooperativity applies only to the nearest neighbors, by including hydrogen bonds that are farther away. The asymmetric kinetic effect of the orientation of a given base-pair may extend well beyond the nearest neighbors. Observations that support the relaxation of our nearest-neighbor assumption include experiments where pairs of base-pairs in duplex DNA has been shown to interact across a distance of the order of a few nanometers (electronic coherence length), about an order of magnitude larger than the distance between two neighboring base-pairs^35,36^. When the kinetic interaction extends beyond the nearest neighbors, it becomes possible for only a few percent of $GC$ skew to set the unzipping orientation during DNA replication.

As shown in the Fig. 9, there are two types of interfaces between two replichores: (a) As we move from the 5′-end of a strand towards its 3′-end, a $G$-dominant replichore changes to $C$-dominant one at the interface (bottom strand, right interface), or, (b) a *C*-dominant replichore changes to $G$-dominant one at the interface (bottom strand, left interface). The kinetics of bonding/dissociation of base-pairs at these two types of interfaces are entirely different. This difference has to do with the direction of the catalytic arrows of the base-pairs on either side of the interface. The arrows in the middle of the two strands in Fig. 9 show the direction of the catalysis, which is determined by the sign of the $GC$ skew. For the type of interface, mentioned in (a) above, the asymmetric cooperativity changes from left mode to right mode as we move towards left, and the catalytic arrows point at each other, as in the first interface from the right of the strand in Fig. 9, denoted with a red dot. The hydrogen bonds of base-pairs at the interface will have their barriers lowered due to catalytic influence from the neighboring base-pairs in both left and right directions, and are prone to dissociate easily. This explains the reason behind the function of replichore interfaces of type (a) as origins of replication. On the other hand, in type (b), the catalytic arrows point away from each other, as in the first replichore interface from the left of the strands in Fig. 9. This results in the kinetic barriers of the hydrogen bonds of base-pairs at the interface to be raised, and thus results in such interfaces to function as replication termini. It is easy to understand that, higher the $GC$ skew, higher will be the sequence-dependent asymmetric cooperativity, and consequently, higher will be the rate of unzipping and hence of replication. It is interesting to note that such a correlation between the magnitude of skew in a genome and its replicative speed has already been observed^37^.

The other pair of nucleotides, $A$ and $T$, are also observed to be asymmetrically distributed across the two strands of DNA in various genomes, and its switch is correlated with replicative origins^24^. But the base-pair orientation does not consistently correlate with the direction of replication across genomes of different organisms^37^, like that of the $GC$ base-pair. For example, $T$ is enriched on the leading strand in *Human* genome, whereas $A$ is enriched on the leading strand in *B*. *Subtilis* genome. It is possible that different environmental factors dictate the asymmetric cooperativity mode of the base-pair. We would like to emphasize that, while the directionality of the unzipping machinery is determined by the *GC* skew within this picture, the direction of new strand synthesis would still be dictated by the 3′–5′ directionality of the template strand, due to our assumption of weaker sequence-dependent asymmetric cooperativity, compared to strand directionality-dependent asymmetric cooperativity.

### Asymmetric primer extension kinetics

**Figure 10:**
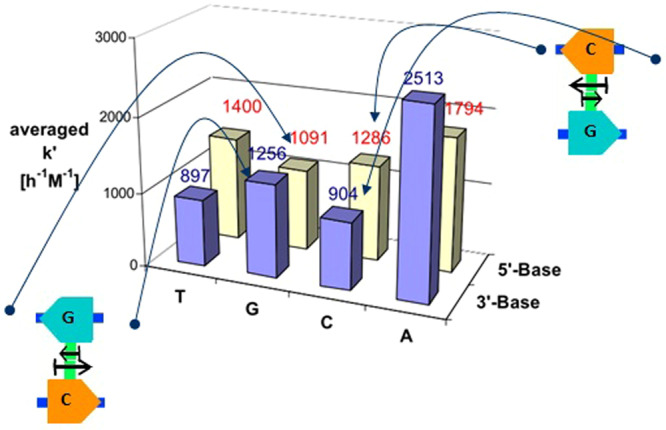
The bar plot from^13^, reproduced here with permission from PNAS, shows the experimentally observed dependence of rate of extension of a template-attached primer by a single nucleotide on its neighboring nucleotides. The rate of extension is higher when the nucleotide *G* is the immediate neighbor on the 3′ side of the primer, or when *C* is the 5′ side immediate neighbor on the downstream binding nucleotide. With *G* at the 5′ side and *C* at the 3′ side, the rates are lower. In other words, the base-pair 5′–*C*–3′/3′–*G*–5′ supports higher rate of nuceotide incorporation to its left compared to 5′–*G*–3′/3′–*C*–5′, whereas 5′–*G*–3′/3′–*C*–5′ supports higher incorporation rate to its right compared to 5′–*C*–3′/3′–*G*–5′. This experimental observation strongly supports our view that the orientation of the base-pair dictates the direction of asymmetric cooperativity. The mode of asymmetric cooperativity also agrees with the direction of catalysis and stabilization derived from the relationship between replication directionality and *GC* skew sign. The asymmetric cooperativity also seems to exist in *AT* base-pair, but with an added constant component when the nucleotide *A* is present on the replica strand neighborhood. This figure is excluded from CC BY license that applies to the rest of the article and requires permission from PNAS to reproduce.

An important experimental source of support for the connection we established above between the asymmetric cooperativity mode and the orientation of the base-pairs, i.e., 5′–*G*–3′/3′–*C*–5′ versus 5′–*C*–3′/3′–*G*–5′, is provided in^13^, where the kinetics of *non-enzymatic* primer extension (which includes both hydrogen and covalent bonding) is measured as a function of various sequence neighborhoods. The asymmetric influence of a hydrogen bond on the incorporation kinetics of a monomer nearby is illustrated in Fig. S6 of^13^, and reproduced with permission here in Fig. 10. First, the rate of incorporation of a nucleotide is shown to be dependent on the type of nucleotide present on the 3′ and the 5′ neighboring ends of the incorporated nucleotide (Table 1 of^13^). Second, the rate of incorporation depends on the orientation of the neighboring base-pairs, i.e., 5′–*G*–3′/3′–*C*–5′ versus 5′–*C*–3′/3′–*G*–5′. For example, 5′–*C*–3′/3′–*G*–5′ supports higher rate of nuceotide incorporation to its left compared to 5′–*G*–3′/3′–*C*–5′, whereas 5′–*G*–3′/3′–*C*–5′ supports higher incorporation rate to its right compared to 5′–*C*–3′/3′–*G*–5′ (please see Fig. 10). Third, the direction of asymmetric enhancement (5′–*C*–3′/3′–*G*–5′ catalyzing the *left* neighbor) of the incorporation rate agrees with the direction of catalysis that we arrived at from the well-established relationship between the direction of unzipping during replication and $GC$ skew.

### Palindrome and inverted repeat instability

Special sequences, whose bottom strand sequence is the reverse of the top strand sequence, exhibiting a special kind of symmetry called “dyad symmetry”, are called palindromes. An example is the sequence 5′–*CTAG*–3′/3′–*GATC*–5′, which has been shown to be extremely rare in bacterial genomes^38^. Perfect palindromes are generally under-represented in most genomes^39^, and have been shown to be fragile^40^. Inverted repeats are sequences with an intervening sequence between the two symmetric “arms” of a palindromic sequence. As with the larger-scale approximate dyadic symmetry of the $GC$-skew-switching locations leading to origins of replication, these smaller-scale dyadic symmetry elements too serve as origins of replication and transcription^41^, and function as targets for restriction enzymes^42^. Within our model, these properties follow from the increased symmetry of palindromic and inverted repeat sequences.

**Figure 11:**
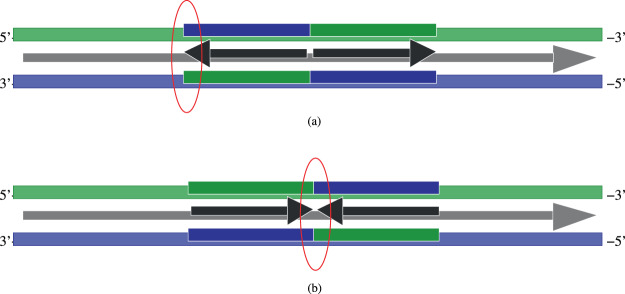
Schematic diagram illustrating the emergence of palindromic instability from sequence-dependent asymmetric cooperativity. The figure illustrates the effect of insertion of a palindromic sequence within a single replichore with a right asymmetric cooperativity mode. Due to the dyadic symmetry of a palindromic sequence, the asymmetric cooperativity modes of the two arms of the palindromic sequence point in opposite directions. The hydrogen bonds at an interface where the two asymmetric cooperativity arrows point at each other will have their kinetic barriers reduced, weakening the bonds and resulting in localized unzipping. There are two types of palindromic sequences: (**a**) When two the asymmetric cooperativity modes from the two arms of the palindrome point away from each other, one of the two ends of the palindrome become unstable due to the change of cooperativity mode. This end is highlighted with a red ellipse in the figure. (**b**) When the modes from the two arms are such that the arrows point at each other, the bonds at the center of the palindrome will weaken, leading to local unzipping and cruciform extrustion. These locations on the genome are vulnerable to break, due to the instability introduced by localized unzipping.

The dyadic symmetry of the palindromic sequences, illustrated in the Fig. 11, causes the asymmetric cooperativity modes of the two arms of the palindrome to point in opposite directions. This results in two possibilities: (a) The two asymmetric cooperativity arrows of the two arms point away from each other, or (b) The two arrows point at each other. The former case, shown in the Fig. 11(a), makes the center of the palindrome to behave like a replication terminus (see also Fig. 9), but at one of the ends of the palindrome, the two arrows point at each other, rendering that location to be unstable. This location is denoted by a red ellipse in the Fig. 11(a). In the second case, the two arrows point at each other in the middle of the palindrome, resulting in instability at the center of the palindrome. This instability can lead to local unzipping at those locations, and in case (b), may allow for the formation of secondary structures such as cruciform extrustion. Inverted repeats, which have an intervening sequence between the two arms of a palindrome, will also lead to local instability, due to the $GC$ skew of the intervening sequence being different in direction from the skew of one of the arms of the palindromic sequence that contains it. This clear separation of palindromes into two different types (a) and (b) provides a possibility to experimentally verify our hypothesis of sequence-dependent asymmetric cooperativity. Since the fragile locations, where the double strand is unstable with respect to thermal fluctuations, are different in the two types, a bioinformatic/experimental search for fragile locations in these two types can provide clear evidence for or against our hypothesis.

### Polar inhibition of replication forks

Another source of experimental support is the documented asymmetric (polar) and sequence-dependent rate of movement of the “unzipping machinery” (the replication fork) as it traverses the genome during replication. During DNA replication, the replication fork moves unidirectionally from the origin of replication, with the direction correlated with the *GC* skew sign. Thus, stretches of genome with $G$-enriched on one strand should allow the fork to proceed in one direction, while inhibiting its movement in the opposite direction. Such polar inhibition of replication forks through *G*-enriched sections has been experimentally observed^43–45^, and are usually explained as due to triple-helix formation, although there has been no direct experimental evidence for the triple-helix formation, *in vivo*. This sequence-dependent unidirectional movement of replication fork arises from the asymmetric kinetics of (un)zipping of the asymmetrically cooperative DNA, within our model. It has to be noted that the permissive and blocking directions set by $GC$ skew are consistent for the movement of both the DNA unzipping machinery and the replicative and transcriptional machinery through $G$-enriched sections of different genomes. Thermodynamic parameters of DNA unzipping alone cannot capture such direction-dependent rates of movement of the replication fork. More support for sequence-dependent unidirectional movement of the replication fork are a) the direction-dependent slowdown of the replication fork at transcription-start and stop elements^46^, b) the direction-dependent pause or termination of replication at *ter* elements of *E*. *Coli*^47^, with the choice between pause and termination determined by the speed of the replisome^48^, and c) genetically-determined replication slow zones in budding yeast^49^ and *D*. *Melanogaster*^50^ genomes. At the single-molecule level, the orientation of the terminal base-pair of DNA hair-pin molecules has been discerned using kinetics of unzipping through a nanopore^51^. More recently, the differences in lifetimes of stacking interactions between swapped-sequence pairs such as 5′–*CG*–3′/3′–*GC*–5′ and 5′–*GC*–3′/3′–*CG*–5′ have been shown to span several orders of magnitude^52^, further supporting our hypothesis of the connection between base-pair orientation and kinetics.

### Asynchronous replication of mammalian mitochondria

Mammalian mitochondrial DNA replicates slowly compared to the rates of replication of prokaryotes such as *E*. *Coli*, and appears to have minimal evolutionary pressure for rapid replication^53^. In the absence of such pressure, the mitochondrial genome is not constrained to simultaneously replicate independent segments, and has been shown to undergo a different mode of replication (called Strand Displacement Model), where the two strands replicate independently, successively, and asynchronously^53^. This mode of replication avoids employing lagging strand synthesis to replicate major sections of the genome and thus foregoes the complications associated with it. The $GC$ skews of these mammalian mitochondrial genomes are larger in magnitude and never cross zero^54^, implying that the asymmetric cooperativity mode remains the same for a major portion of such genomes, within our picture. This suggests that, under minimal evolutionary pressure for faster replication, mammalian mitochondria have dispensed with the lagging strand synthesis approach, and adopted a $GC$ skew profile that supports the continuous replication of both the strands.

## Falsification approaches

The model above and its central premise, that of the presence of sequence-dependent asymmetric cooperativity in DNA, can be experimentally verified or falsified with currently available technologies. The relationship between $GC$ skew and asymmetric kinetic barriers on the two sides of a double strand DNA can be tested thoroughly by unzipping a single dsDNA molecule using Atomic Force Microscope from both ends and documenting the force signatures, as has been done here^55^, taking care to do the experiment near equilibrium conditions. According to our model, it should be easier to unzip the sequence 5′–(*C*)_*n*_–3′/3′–(*G*)_*n*_–5′ from the left end and 5′–(*G*)_*n*_–3′/3′–(*C*)_*n*_–5′, from the right end, in an environment resembling *in vivo* conditions of prokaryotic genomes.

Sequence-dependent asymmetric cooperativity can be quantified by varying the sequence and measuring the difference in the forces required to unzip the dsDNA molecules from the left and right ends. Also the model’s assumption that only nearest neighboring base-pairs affect the kinetics of unzipping can be tested and modified as necessary. The connection between origins of replication and asymmetric cooperativity can be tested by working with sequences whose $GC$ skew switches between negative and positive values and measuring the lifetimes of hydrogen bonds of base-pairs at the switching location, through NMR experiments, taking care to include the helicity and the topology of the strands as influencing variables. The hydrogen bond lifetimes at the switching location should be lower when the skew switches from $G$-dominant to *C*-dominant, and should be higher when the switch is the other way around, when the environmental variables are kept at values similar to those observed in prokaryotic genomes.

Another falsification approach, using either bioinformatics or experiments, is to verify the presence of two types of palindromic sequences, type (a) and type (b), as explained above. The fragile locations on these two types of sequences would be different, according to the model. Type (a) palindromic sequences would have fragile locations at one of their ends, whereas, type (b) sequences will have fragile locations at the center of the palindrome.

## Discussion

We have shown that some fundamental structural and functional elements of DNA can be connected to the presence of asymmetric cooperativity in DNA. Asymmetric cooperativity, defined as an unequal and non-reciprocal kinetic influence between two interstrand hydrogen bonds, necessitates breaking of left-right symmetry of monomers, resulting in directional monomers and strands, denoted in the biological literature as 3′–5′ directionality. In this article, we factorized asymmetric cooperativity into sequence-independent and sequence-dependent parts, operative in single and anti-parallel double strands respectively, for ease of analysis. We have argued that anti-parallel strand orientation of DNA enables independent unzipping and replication of multiple segments of DNA simultaneously, from predictable origins of replication (for prokaryotes), through sequence-dependent asymmetric cooperativity, since the stronger sequence-independent part is cancelled due to the anti-parallel orientation of the two strands of the duplex. Such a replicative organization would result in substantially shorter replication time for self-replicating heteropolymers with anti-parallel strands, when compared to heteropolymers with parallel strands. The latter’s unzipping direction would be set by the parallel strands themselves through sequence-independent asymmetric cooperativity, is therefore frozen along the entire length of the strands and cannot be altered to achieve simultaneous replication of independent segments, within our model. Parallel-stranded DNA have been shown to readily form, given appropriate sequences, under physiological conditions, *in vitro*^7–10^. There is also evidence of formation of parallel-stranded RNA sequences *in vivo* in gene-silencing experiments^6^. Thus, biochemical implausibility of formation of the parallel DNA strands cannot be a reason for the choice of anti-parallel strands. Experiments comparing the thermodynamic stabilities of anti-parallel and parallel-stranded DNA have shown that the former are more stable, and have higher melting temperatures^7,8^. This stability is essential for DNA to preserve information across multiple generations, which is achieved by raising the thermodynamic barrier for the double-strand to single-strand (helix-coil) transition, thereby reducing the time spent by DNA in the mutationally more susceptible single-stranded state. However, in the primordial scenario we are interested in, such high thermodynamic barriers are counterproductive, since that would prevent the separation of daughter strand from the template in time to start the next round of replication^11^, making anti-parallel strands a replicatively less favorable choice. Evolution appears to have overcome these competing requirements of high and low thermodynamic stabilities of double-stranded anti-parallel DNA by utilizing sequence-dependence of thermodynamic and kinetic barriers for helix-coil transition. This sequence-dependence enables predictable sections of DNA with low barriers to function as origins of replication, which in turn provide access to thermodynamically more stable sections of DNA through cooperative unzipping.

We showed that sequence-dependent asymmetric cooperativity cannot be instantiated in anti-parallel strands with homomolecular inter-strand bonds, due to the absence of left-right asymmetry of the homomolecular base-pair. This necessitates the introduction of heteromolecular inter-strand bonds, which possibly led to *G*/*C* and *A*/*T* heteromolecular inter-strand bonding. We argue that unzipping directionality during replication is set by asymmetric nucleotide composition or $GC$ skew, the excess of one nucleotide over another over the entire segment of DNA over which the unzipping machinery moves in the same direction. This provides an evolution-based rationale for the existence of asymmetric nucleotide composition in genomes, otherwise detrimental due to the consequent reduction of protein-coding space. Our identification of $GC$ skew as the cause of unzipping and replication directionality, instead of an effect of the latter, through sequence-dependent asymmetric cooperativity, also helps us make sense of the nature of sequences at replication origins. These sequences at replication origins usually exhibit an approximate dyadic symmetry, prominent example being palindromic sequences. We have shown that due to the switching of asymmetric cooperativity modes from right to left, the hydrogen bonds at these locations have lowered kinetic barriers, and hence can break easily during thermal fluctuations, enabling them to function as origins. Similar arguments apply for sequences at the replication termini, where the kinetic barrier is raised due to inhibitory kinetic influence from either side of the $GC$ skew-switching location.

We speculate that the kinetics of unzipping underlie information-encoding mechanism in genomes^56^, with thermodynamics playing a more subdued role. We have referred to multiple experiments and observations that point to the existence of asymmetric cooperativity in DNA. We have also included possible experimental tests to validate the proposed connections, where appropriate. Importantly, our theoretical picture might make it possible to decipher the connection between DNA sequence and its propensity and rate of unzipping under various cellular environments, by going beyond thermodynamic analyses alone, thereby throwing a clearer light on the mechanisms governing the specific genomic response to these cellular environments. These connections thus also provide possible means of manipulating the genomic responses through rational alteration of local sequences, informed by the inclusion of sequence-dependent asymmetric cooperativity. Crucially, by linking together DNA sequence and its rate of replication, asymmetric cooperativity might have made prebiotic evolution possible in the first place. In conclusion, asymmetric cooperativity, if experimentally verified to be present in DNA, can provide a unifying theoretical picture within which the evolutionary rationale for the existence of some fundamental properties of DNA can be understood.

A reasonable counter-argument against the foregoing is the absence of any evidence of temporal parallelization of replication in the possibly more primordial RNA-based life forms, such as dsRNA viruses, as a reviewer has pointed out. The genomic organization of RNA-based genomic systems of viruses appear to be dictated by the thermodynamic instability of long RNA molecules^14^, and less by the evolutionary pressure towards high rate of replication. The manufacture of the capsid proteins of RNA viruses inside their hosts has been shown to be the rate-limiting step during the viral replication^57^, which reduces the evolutionary pressure on the RNA genomes to replicate faster. RNA viruses increase the information content of their genomes, subject to the constraint on the length of RNA molecules, by dividing their genomes into multiple, small, unconnected RNA strands, called segments, that replicate unidirectionally, asynchronously and independently of each other^14,15^. The absence of evidence for RNA-based genomes with replichore-based genomic organization similar to that of DNA is also possibly due to the current environmental conditions on Earth being different from the ones prevailing during the “RNA-world” scenario which possibly supported longer RNA molecules^16,17^.

### Limitations of the model

As with nearly all biophysical models, the model constructed above is very much an abstraction of the real processes inside DNA, which leaves out a vast majority of other interactions. A more realistic model, while including all interactions, say between DNA and the replisome proteins, would be hopelessly complicated to be amenable to such simple theoretical arguments. In isolating one particular interaction to study in detail, namely, the influence of neighborhood on the kinetics of hydrogen bonding, we have ignored the influence of other related degrees of freedom of DNA, such as its helicity or topology, on our subsystem of study. The interactions between these other degrees of freedom and asymmetric cooperativity would be crucial to understand higher order functions, such as the influence of negative supercoiling or superhelicity on replication and transcription origins, for instance. Another technical limitation is our assumption that only nearest neighbors influence the kinetics of hydrogen bonds, which can be safely relaxed without jeopardizing our conclusions. Although we have justified our exclusion of interactions of DNA with other cellular components by situating our study at the time of the evolutionary progenitors of DNA which were not encumbered with such interactions, quantitative analyses of extant systems that go beyond mere understanding require the inclusion of such interactions, for which the above model will merely serve as a simple starting point.
